# Editorial: Silver linings: navigating the challenges and opportunities of organ transplantation in an Ageing population

**DOI:** 10.3389/ti.2026.17841

**Published:** 2026-10-23

**Authors:** Thierry Berney, Annelies de Weerd, Katja Kotsch, Simon Moosburner, Jasper Iske

**Affiliations:** 1 Department of Nephrology, Immunology and Transplantation, Hospices Civils de Lyon, Lyon, France; 2 Department of Surgery, University of Geneva School of Medicine, Geneva, Switzerland; 3 Ilia State University, Tbilisi, Georgia; 4 Division of Nephrology and Transplantation, Erasmus Medical Center Transplant Institute, Rotterdam, Netherlands; 5 Department of General and Visceral Surgery, Charité - Universitätsmedizin Berlin, Berlin, Germany; 6 Department of Cardiothoracic and Vascular Surgery, Deutsches Herzzentrum der Charité (DHZC), Berlin, Germany

**Keywords:** ageing, older donors, older recipients, organ transplantation, senescence

## Introduction

As the world’s population ages, the rising number of older transplant recipients, coupled with the growing demand for organs from older donors, presents significant challenges for transplantation medicine. These challenges require novel considerations for both donor selection and recipient eligibility. While older organs have the potential to help address the shortage of donor organs, they are more frequently discarded due to physiological and immunological factors. Aged recipients, in turn, have been shown to exhibit less pronounced alloimmune responses while being at risk for increased side effects of immunosuppressive therapies and infections. Furthermore, increasing evidence suggests a biological interplay between the graft and the recipient organism affected by age discrepancies. All of the above suggest that aging is one of the most critical risk factors for transplant outcomes and has emerged as one of the fastest-growing research fields in recent years.

In this special issue, we have aimed to explore the multifaceted challenges and opportunities associated with aging populations in the context of organ transplantation. We received a valuable collection of original research articles, reviews, and viewpoints that address aging biology and its relevance to graft function and outcomes, demographic trends and their impact on transplant supply and demand, immunosenescence and its implications for transplant outcomes and rejection risks, age-related changes in immune response and how they affect immunosuppressive therapies, and strategies for optimizing transplantation in older adults, including donor-recipient matching and post-transplant care.

## Older organ transplant recipients

The aging of the general population is a consequence of medical progress and improved standards of living. Older “chronological” age arguably does not necessarily lead to older “biological” age. In parallel, progress in transplant medicine and surgery has allowed us to consider transplantation for an older population with end-stage organ failure who would have been considered too frail in the past. However, how do such patients really benefit from organ transplantation? How can we balance the ethics of organ donor shortage versus the naturally lower life expectancy of patients, who are nevertheless equally entitled to the best possible healthcare?

In a registry review from Germany, Schröter et al. analyzed the outcomes of kidney transplantation, particularly infection burden, graft function, and mortality, in a cohort of 572 patients stratified by age. In older recipients (>60 years old), the unadjusted 5-year rates of graft failure, mortality, and infections were significantly higher, both overall and for specific types, including pneumonia, urinary tract infections, invasive opportunistic infections, and multidrug-resistant infections. However, after adjustment for confounding variables, only the 5-year mortality rate was, unsurprisingly, higher in the older age group. Older patients were more likely to have *Pseudomonas aeruginosa* infections, more severe herpesvirus infections, and higher infection-related morbidity. This study demonstrates that age alone is not a predictor of adverse events, but that post-transplant care for older recipients should focus on early infection control and pathogen-targeted surveillance.

Fayos et al. focused on infection after kidney transplantation in older recipients in a prospective single-center cohort study of 712 patients over 8 years. The occurrence of overall, bacterial, and opportunistic post-transplant infection was investigated, stratifying by 10-year age groups, including those aged >80 years. In unadjusted analyses, each 10-year increase was associated with an increase in the incidence of overall, bacterial, and opportunistic infections. After multivariate adjustment for confounders, this association remained significant for overall and bacterial infections, with the highest risk observed in patients >70 years. As a potential explanation for this observation, the authors reported a decrease in circulating T-cell counts (CD3, CD4, and CD8) with each 10-year increase in age. Fayos et al. thus suggest, unlike Schröter et al., that the infection risk after kidney transplantation increases with age, particularly above 70 years. These differences may be explained in part by the higher age of their cohort and differences in follow-up.

In a retrospective, single-center study of >1700 kidney transplant recipients, Winkler et al. analyzed and stratified causes of graft loss by era and by recipient age. Unsurprisingly, the proportion of death-with-function increased stepwise with age. The reverse was observed for rejection as a cause of graft failure, with its incidence markedly decreasing with increasing age. Similar observations were made in the three time periods studied between 1995 and 2022.

## Older organ donors

The aging of the general population also affects organ donor age, which has been increasing in recent decades. The upper age limits for considering a donor as “marginal” or an “expanded criteria donor” have had to be raised to face this new reality. An age-adjusted donor score has been proposed to facilitate the selection of organ donors aged >65 years [1]. Intriguing reports have shown that chronic antibody-mediated rejection is more common in recipients of kidneys from older donors [2]. In the context of living donation, we already know that a kidney transplant from an older donor is preferable to waiting for a younger donor with brain death (DBD) [3]. But how does this function in practice for deceased donation? How does risk aversion manifest when an organ from an older patient is offered? What are the age limits? Are there mitigation strategies?

An article by Chotkan et al. examined the causes of declining donor offers in the Netherlands and analyzed all offers made by the national organ allocation system between 2018 and 2023. During this 5-year period, the donor age progressively increased from a mean of 54 to 57 years, with the proportion of donors >65 years reaching 36%. Concurrently, the proportion of declined donor offers has increased from 23% to 30%. In multivariate analysis, older age (>65 years) was found to be one of the main factors independently associated with declining a donor offer. The other factors were donation after circulatory death (DCD) and comorbidities (hypertension and diabetes). This suggests that older donors are still considered “marginal” independently of other potential medical considerations and without necessarily solid evidence of poorer quality.

The Eurotransplant Senior Program (ESP) was established to improve kidney allocation from older donors and address the shortage of organs for kidney transplantation. The ESP allocates kidneys from deceased donors aged ≥65 years to older recipients ≥65 years who leave the general kidney waiting list in exchange for significantly shorter waiting times. The program is largely based on minimizing cold ischemia time (CIT) by allocating organs locally. An opinion piece by Giorgakis et al. argued for applying an ESP-like age-matching scheme within U.S. allocation rules. The authors convincingly suggested that such realignments could reduce organ discard rates, enhance long-term graft utility, alleviate the burden of decision-making on patients, and reduce the need for re-transplants in younger patients.

An article by Langer et al. examined the outcomes of patients transplanted under this scheme. One-year and 5-year graft and patient survival rates were excellent and notably superior to survival rates for patients on dialysis. Delayed graft function (DGF) was high, at nearly 30%, but was strongly related to prolonged CIT. HLA mismatch was not related to graft outcomes. An interesting feature of the article is the histology data from biopsies taken immediately after reperfusion. The degree of glomerulosclerosis, interstitial fibrosis, and tubular atrophy (IFTA) was associated with graft failure, and arteriolosclerosis (arteriolar hyalinosis) correlated with a higher risk of primary nonfunction. This study demonstrates the validity of the ESP, emphasizes the importance of maintaining short CIT in this donor population, and suggests that pre-transplant biopsies could provide useful selection criteria for donor acceptance.

The impact of CIT in the ESP was further studied by Matuschik et al. As mentioned above, within the ESP, kidneys are preferentially allocated locally in order to minimize CIT. In this article, the authors have assessed the impact of CIT on DGF and long-term outcomes in consecutively transplanted ESP recipients from the same donor. DGF incidence was similar for first and second recipients. CIT >12 h significantly increased DGF risk. Death-censored allograft survival was unaffected, but CIT >12 h tripled the risk of mortality. Consecutive transplantation did not disadvantage the second recipient if CIT remains <12 h. CIT >12 h independently predicts DGF and higher mortality, emphasizing the need to minimize ischemia time when older donors are utilized.

Is the detrimental impact of ischemia-reperfusion injury only caused by CIT? In their article, Süsal et al. looked at the impact of ischemic events occurring in the donor before the declaration of brain death. More specifically, the authors examined the impact of cardiopulmonary resuscitation (CPR) in a cohort of ≥65-year-old kidney donors Süsal et al. In this tri-center study from Germany, all kidneys from ≥65-year-old donors (2006–2023) were retrospectively analyzed, and the outcomes of kidney transplants from donors with and without a history of CPR were compared. There were no differences in DGF occurrence or in 1-year or 5-year kidney graft function between CPR and non-CPR donors. Five-year graft and patient survival were similar. Although some degree of selection bias is unavoidable in retrospective studies, the data indicate that ≥65-year-old DBD donors with a history of CPR are potentially suitable for kidney transplantation without negative impact on outcomes.

Maanaoui et al. investigated the outcomes of kidney transplantation from what the authors refer to as “very old” donors. More specifically, they studied the risk factors for long-term graft loss or DGF after transplantation of kidneys from DBD donors >70 years. In a French national cohort of >1,000 recipients, donor hypertension, CIT, and HLA mismatches were significant risk factors for graft loss. With regard to DGF, donor serum creatinine and warm and cold ischemia times were found to be deleterious, while hypothermic machine perfusion had a protective effect. These findings emphasize the paucity of modifiable variables associated with long-term outcomes in older donors and the need for peri-transplant preservation strategies.

## Machine perfusion for organs from older donors

The study by Maanaoui et al. suggested that machine perfusion may be routinely necessary. What about the liver or thoracic organs? Indeed, the routine use of machine perfusion for thoracic organs procured from older donors has been advocated for in ESOT recommendations [4].

Ibrahim et al. surveyed all 39 liver and kidney transplant centers in Germany, achieving a 56% response rate. Liver transplantation programs utilize both hypothermic and normothermic perfusion, whereas perfusion strategies in kidney transplantation remain largely limited to hypothermic techniques. The majority of liver centers use machine perfusion selectively for marginal grafts and report an average 10% increase in organ utilization rates attributed to machine perfusion. It should be emphasized that, in Germany, where DCD organ donation is not legally permitted [5], advanced donor age is the principal characteristic defining marginal donors.

Giorgakis et al. have conducted a national cohort study using registry data from the United States and examining the activities and outcomes of various machine perfusion techniques in liver transplantation according to donor age. The cohort showed accelerated growth in DCD liver transplantation after FDA approval of machine perfusion. Donors ≥60 increased 7.8-fold, including those ≥70. By 2025, DCD livers accounted for 43% of transplants, with 61% from donors ≥50. The use of normothermic regional perfusion (NRP), hypothermic machine perfusion (HMP), and sequential NRP-HMP increased significantly. This has led to a decrease in the average waiting time without impacting graft or patient survival. This study shows that utilization of machine perfusion techniques is associated with an increased utilization of older DCD liver transplants and an acceptable early graft survival.

## Senotherapeutics: a look into the (near) future

Chronological age captures organ quality only imperfectly, and markers that reflect biological rather than calendar aging are therefore increasingly sought as the donor pool grows older. In a review closing this special issue, Buch et al. examined the role of cellular senescence, a hallmark of aging, in the successive stages of the transplant process, from procurement and preservation to graft function after implantation. The authors argued that senescence burden could serve both as an objective metric of organ quality and as a therapeutic target in its own right. Of particular interest is the proposed combination of senotherapeutic agents with *ex vivo* machine perfusion, which would allow senescence burden to be quantified and targeted interventions to be delivered to the graft before implantation, with the aim of reconditioning marginal organs rather than discarding them. These strategies remain largely experimental, but they illustrate how the assessment of donor organs may move beyond age alone in the future.

## Silver linings

Taken together, the contributions to this special issue draw a more nuanced picture than the one that has long guided practice. Outcomes are driven by a variety of modifiable (infection burden, cold ischemia time, donor comorbidity, and preservation strategy) and unmodifiable risk factors. Age is a powerful risk marker but, unfortunately, it is not a modifiable one. Older recipients derive a survival benefit from transplantation that dialysis cannot match; organs from older donors perform considerably better than their discard rates would suggest; and age-aligned allocation, short ischemia times, pre-implantation histology, and machine perfusion all offer concrete ways of converting a demographic constraint into additional transplants. The task in the coming years will be to replace chronological age with measures of biological age that are robust enough to guide acceptance decisions at the bedside. Should we succeed, the aging of our donor and recipient populations may present more opportunities than threats, which is the silver lining we had in mind when assembling this collection.
